# Kidney Segmentation from Dynamic Contrast-Enhanced Magnetic Resonance Imaging Integrating Deep Convolutional Neural Networks and Level Set Methods

**DOI:** 10.3390/bioengineering10070755

**Published:** 2023-06-24

**Authors:** Moumen T. El-Melegy, Rasha M. Kamel, Mohamed Abou El-Ghar, Norah Saleh Alghamdi, Ayman El-Baz

**Affiliations:** 1 Electrical Engineering Department, Assiut University, Assiut 71515, Egypt; 2 Computer Science Department, Assiut University, Assiut 71515, Egypt; 3 Radiology Department, Urology and Nephrology Center, Mansoura University, Mansoura 35516, Egypt; 4 Department of Computer Sciences, College of Computer and Information Science, Princess Nourah Bint Abdulrahman University, P.O. Box 84428, Riyadh 11671, Saudi Arabia; 5 Bioengineering Department, University of Louisville, Louisville, KY 40292, USA

**Keywords:** DCE-MRI, kidney segmentation, U-Net, level set

## Abstract

The dynamic contrast-enhanced magnetic resonance imaging (DCE-MRI) technique has taken on a significant and increasing role in diagnostic procedures and treatments for patients who suffer from chronic kidney disease. Careful segmentation of kidneys from DCE-MRI scans is an essential early step towards the evaluation of kidney function. Recently, deep convolutional neural networks have increased in popularity in medical image segmentation. To this end, in this paper, we propose a new and fully automated two-phase approach that integrates convolutional neural networks and level set methods to delimit kidneys in DCE-MRI scans. We first develop two convolutional neural networks that rely on the U-Net structure (UNT) to predict a kidney probability map for DCE-MRI scans. Then, to leverage the segmentation performance, the pixel-wise kidney probability map predicted from the deep model is exploited with the shape prior information in a level set method to guide the contour evolution towards the target kidney. Real DCE-MRI datasets of 45 subjects are used for training, validating, and testing the proposed approach. The valuation results demonstrate the high performance of the two-phase approach, achieving a Dice similarity coefficient of 0.95 ± 0.02 and intersection over union of 0.91 ± 0.03, and 1.54 ± 1.6 considering a 95% Hausdorff distance. Our intensive experiments confirm the potential and effectiveness of that approach over both UNT models and numerous recent level set-based methods.

## 1. Introduction

Chronic kidney disease (CKD) is a critical public health concern whose incidence rates are rising rapidly around the world. It is characterized by heterogeneous disorders causing a change in kidney structure and progressive loss in its function [1,2]. Early diagnosis of CKD is essential to avoid total renal damage and consequently save patients’ lives. Traditional medical acts such as blood tests and urine sampling are not effective for evaluating kidney function since they can detect failure after losing almost all of renal function. Biopsy, the gold-standard technique, is also not effective since it is expensive, time-consuming, and may cause bleeding in or pain to patients. Recently, DCE-MRI has been explored as a noninvasive technique that is able to provide information about kidney anatomy and functionality [3,4].

**Figure 1:**
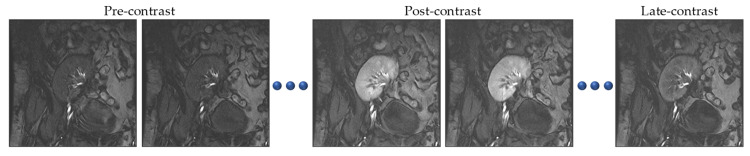
Sample DCE-MRI scans of one subject visually reflecting the effect of the injected contrast agent on the kidney.

In the DCE-MRI technique, a contrast agent is injected into the patient’s blood stream, and during the perfusion, successive images are quickly acquired for the kidney [3]. Accordingly, we have a dataset of about 80 varying-contrast DCE-MRI scans per patient (see Figure 1). Precise segmentation of kidneys from acquired images is a prerequisite in analysis pipelines. However, kidney motions and intensity variations as well as the low spatial resolution of images are considered major obstacles to performing this task. Manual segmentation of kidneys from DCE-MRIs is an inefficient, labor-intensive, and time-consuming process. Toward this end, over the years, automatic DCE-MRI kidney segmentation has been subject to extensive search.

Related Work: Deep learning is a machine learning technique that imitates the learning process of the human brain and extracts features from data in an unsupervised manner. Convolutional neural networks (ConvNets) are a subset of deep learning that is having an increasingly important role in the segmentation of the human organs from medical scans [2,5]. Several studies have been conducted to accomplish kidney segmentation from CT images. Yet, rather limited work has addressed the segmentation of kidneys from MRI images [6,7,8,9,10,11,12,13,14,15,16,17]. For instance, Lundervold et al. [6] developed ConvNet based on transfer learning from a brain hippocampus labeling problem to the segmentation of kidneys via 3D DCE-MRI. Haghighi et al. [7] employed two UNT models [8] to perform localization and segmentation tasks for kidneys on 4D DCE-MRI data. The authors in [9] introduced two different ConvNet-based approaches for automatic kidney segmentation from MRI scans. While Brunetti et al. [10] used a genetic algorithm with ConvNets to perform kidney segmentation, Milecki et al. [11] employed thresholding techniques and morphological operators with ConvNet for the same reason. Isensee et al. [12] used a nnUNT model for abdominal organ segmentation, including both kidneys, from MRI images and achieved the top accuracy in the CHAOS challenge [13]. Asaturyan et al. [14] used 3D Rb-UNT and 3D FC-DenseNet [15] models for localizing and segmenting kidneys from DCE-MRIs. Later on, Goyal et al. [16] used the well-known Mask R-CNN architecture [17] for automatic kidney segmentation in MR images and employed morphological operations to improve its segmentation performance.

Summarizing the main features of the most closely related works, Table 1 reports their attained segmentation accuracy measured by the Dice similarity coefficient (DS). In Table 1, IP/OP denotes in-phase/opposed-phase, while SPIR refers to spectral pre-saturation inversion recovery.

As reported in the table, the topmost segmentation accuracy was achieved by Isensee et al. [12]. However, this accuracy is that of the multi-organ segmentation task, and the particular kidney segmentation accuracy is not given [12]. The highest reported kidney segmentation accuracy was achieved by Brunetti et al. [10] with a DS score of 91% on datasets of 18 patients. While the reported segmentation results seem promising, they are not excellent. The main reason behind this is that ConvNets generally necessitate a sufficiently large amount of training data, a requirement that is difficult to satisfy in the medical field. In almost all the works reported in Table 1, the number of patients is no larger than 40. The numbers of patient datasets in [14,16] are apparently larger. Nonetheless, the numbers of MRI slices in both of them do not exceed 2423.

We, in this paper, develop another deep learning approach for automatic kidney segmentation from DCE-MRI data. Motivated by its success in segmentation tasks of medical data, our approach relies on the UNT architecture and its variants [18,19]. We train, validate, and test our approach on real DCE-MRI datasets from 45 patients, with each dataset having approximately 80 MR slices. We perform data augmentation in several ways, thus increasing the number of training MR images to more than 40 thousand; i.e., it is considerably larger than that in all the reported works in Table 1. We carry out several ablation experiments to analyze and tune up the proposed deep models. For evaluating the deep models, we use three statistical evaluation metrics: the Dice similarity coefficient (DS), intersection-over-union (IU), and 95% Hausdorff distance (HD95%) [20,21]. Our experiments successfully demonstrate a DS score better than 0.94, which is higher than that in the previous works.

Seeking even better performance, we analyze the potential of the proposed deep approach. Our analysis indicates that the deep model does not capture well the kidney’s shape information, which is of paramount importance in the segmentation process. The learning of such information by the deep network model would require larger datasets. As a solution, we resort to level set (LST) methods [22] as a popular segmentation technique in the medical domain, which is flexible enough to easily accommodate shape information in its formulation. According to the literature, incorporating such important information into the LST method [23,24,25,26,27,28,29,30,31,32,33,34] results in significant segmentation accuracy in kidney segmentation.

More specifically, we extend our proposed approach to a two-phase DCE-MRI kidney segmentation one, named the UNT Level Set approach (UNLS). In the first phase, we employ the deep UNT-based model to predict a kidney probability map for a DCE-MRI image. In the second phase, a LST method is formulated to minimize a new energy functional that considers both the predicted probability map and the kidney’s shape prior (SHP) information, thus leading to the final kidney segmentation.

Evaluating the UNLS approach on the same DCE-MRI datasets, it achieves a higher DS score of more than 0.95. The performance boost is even more evident from the HD95% metric with a gain as high as 8.76 mm in comparison to that of the original deep model alone. Our extensive experiments affirm the outperformance and reliability of the approach compared to existing kidney segmentation methods in the literature.

The remainder of this paper is structured as follows. Section 2 describes the data used in this work and defines the problem and the notations. Section 3 explains the basic structure of the UNT deep model and its recent variant, the BCD-UNT model, and describes the models training and testing. Section 4 details the proposed two-phase UNLS segmentation approach and reports its experimental results. Finally, the paper is concluded in Section 5.

## 2. Materials and Problem Statement

This section briefly describes the dataset used in this study. It also presents the problem definition and key notations used throughout this paper.

### 2.1. Data

In this work, we utilized real DCE-MRI data belonging to 45 patients who were subject to kidney transplants in Mansoura University Hospital, Egypt. Successive images were acquired using a 1.5 T MRI scanner with phased-array torso coils after injecting each patient with intravenous contrast agent Gd-DTPA at a dose of 0.2 mL/kgBW and a speed of 3–4 mL/s. Each patient’s dataset consisted of about 80 quickly acquired scans of 256 × 256 pixels, which were manually segmented by an experienced radiologist at the hospital. As depicted in Figure 1, the contrast agent passage caused intensity variations over all the images in the sequence, which rendered kidney segmentation more challenging.

### 2.2. Problem Definition and Notations

Overall, each patient’s dataset was composed of a total number of T time point images. Let $I_{t}=\left\{I_{t}\left(x,y\right),\;\left(x,y\right)\in \Gamma ,\;t=1,\ldots ,T\right\}$ be a grayscale DCE-MRI kidney image scanned at a certain time, t, where $I_{t}\;\left(x,y\right)$ is the intensity of the pixel (x,y) in the image domain Γ. For renal function assessment, we needed to separate the kidney from the background in each image in the sequence. That is, we assigned every image pixel (x,y) a label, L={L(x,y), (x,y)∈Γ, L(x,y)∈{K,B}}, such that the pixel either belonged to the kidney region (K) or background region (B).

## 3. Deep UNT-Based Kidney Segmentation Models

Automatic kidney segmentation using deep neural networks has been shown to be promising [35]. The deep UNT model and its amendments are fully ConvNet architectures that have recently drawn considerable attention in the domain of medical image segmentation. Thus, we here employ the standard UNT model and one of its inspired versions dubbed the BCD-UNT model [19].

**Figure 2:**
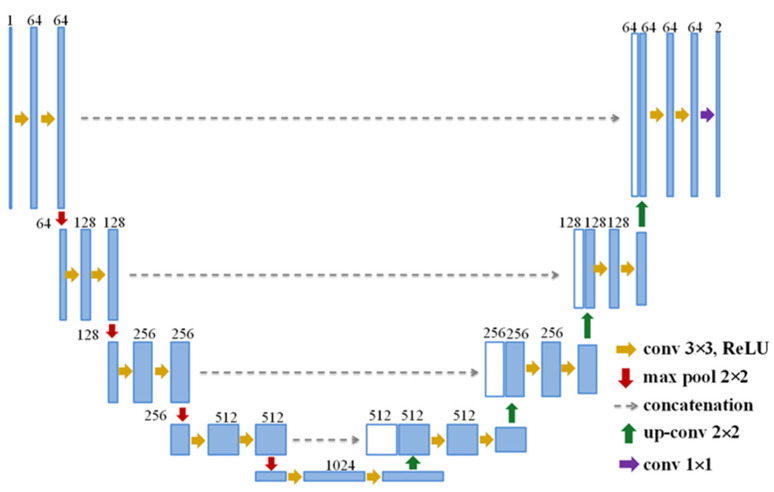
Architecture of the baseline UNT model.

The original UNT model typically consists of two parts, the left side serving as a contracting path and the right side as an expansive path, as shown in Figure 2. Each layer in the contracting path contains two 3 × 3 convolutional layers followed by a dropout layer to prevent overfitting, a rectified linear unit (ReLU) activation function which sets all negative outputs to 0, and a 2 × 2 max-pooling layer that doubles the number of the feature channels and halves the image size. Conversely, each decoder layer has a 2 × 2 up-convolution operation that halves the number of feature channels. Each up-convolved feature map is concatenated with the corresponding feature map from the contracting path. The model ends with a 1 × 1 convolutional layer that uses a sigmoid activation function and produces feature maps of the same size as the input image.

**Figure 3:**
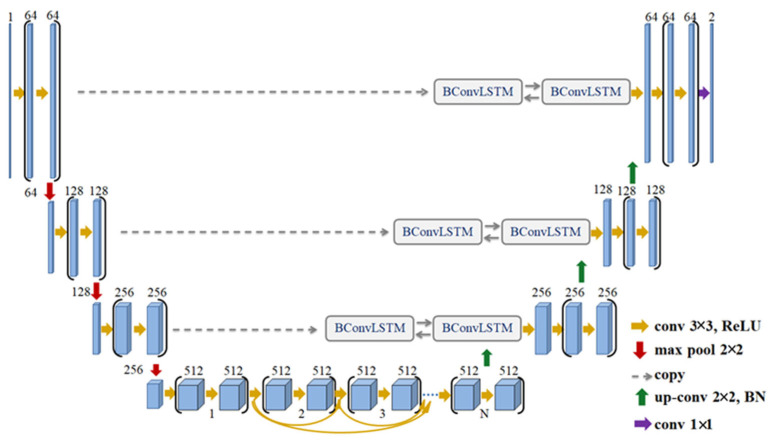
Architecture of the BCD-UNT model.

On the other hand, the BCD-UNT model shown in Figure 3 inherits the advantages of the UNT, bidirectional convolutional long short-term memory (BConvLSTM), and dense convolutions. Each layer in the contracting path of BCD-UNT model consists of two 3 × 3 convolutional filters followed by a ReLU activation function, dropout layer, and 2 × 2 max-pooling layer. In contrast to UNT model, the last convolutional layer of the encoding path in BCD-UNT includes a sequence of densely connected convolutions, in which, feature maps of all previous layers are concatenated with feature map of current layer and used as input for the next convolution. Each layer in the decoding path starts by executing a 2 × 2 up-sampling operation over the previous layer’s output followed by a batch normalization function. The feature maps resulting from the up-convolution operation are combined with the corresponding feature maps of the contracting path employing BConvLSTM. As in the UNT model, a sigmoid activation function is used at the end of the model. In this paper, we employ the BCD-UNT model with three dense blocks.

We trained and validated the models using the datasets of 18 and 12 subjects, respectively, and the other 15 subjects’ data were kept for testing. We performed data augmentation on the training and validation sets. For each image, we applied random translations in x and y coordinates, rotations of (±45°, ±90°, 180°) angles, vertical and horizontal flipping, and zero mean Gaussian noise with (0.01, 0.02, 0.05) variances from the normalized image intensities. As a result, each subject’s dataset was augmented 12 times increasing the number of training images to 16,404 and that of validation images to 10,980.

**Figure 4:**
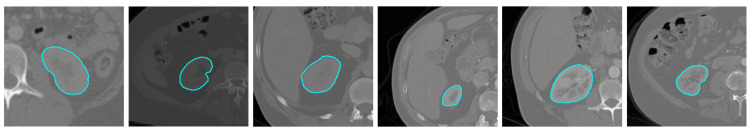
Cropped CT kidney scans for different subjects from KiTS19 challenge dataset with ground truth kidney segmentations shown in cyan.

We further enlarged the training data through the usage of the KiTS19 challenge dataset [36] containing abdominal CT scans of 210 patients with their ground truth segmentations. Each image was manually split into two 256 × 256 sub-images, separately including the left and right kidneys, which increased the number of training images to 40,050. Figure 4 depicts a number of CT images showing the left/right kidneys of different subjects.

### 3.1. Implementation Details

In the training phase, we conducted several trials to tune the parameters of the two models to attain the best possible performance on the validation set. The models were trained for 200 epochs using Adam optimizer and binary cross entropy (BCE) loss function as they are considered the most widely used in medical image segmentation tasks. The initial learning rate (ILR) was set to 0.0001 and was then decayed by 10% every time the validation loss was not lowered for 10 subsequent epochs. In addition, we employed a dropout (DP) with a 50% ratio as a regularization technique to further avert overfitting. The networks were trained in a Python environment using Keras API with a Tensorflow backend. Training was conducted using a workstation with dual 2.20 GHz, Intel Xeon Silver 4114 CPUs, a 128 G of RAM, and two Nvidia GPUs.

### 3.2. Performance Evaluation

**Figure 5:**
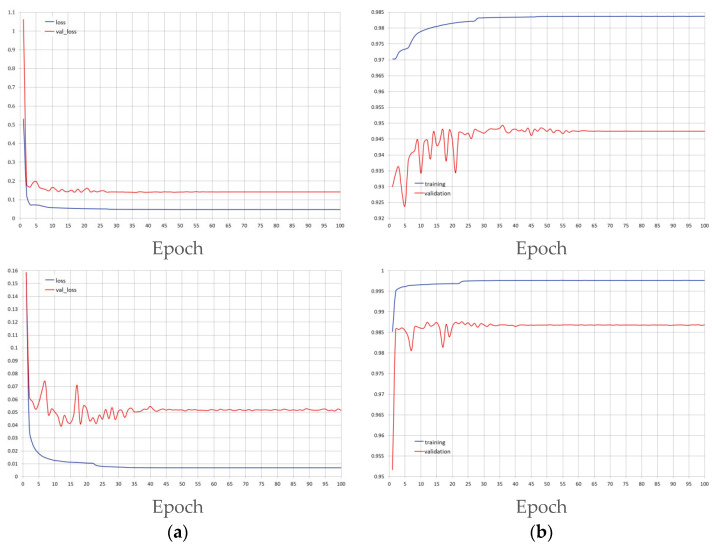
Training and validation BCE loss (**a**) and accuracy (**b**) per epoch for the UNT model (top) and the BCD-UNT model (bottom).

During the training process, we inspected the learning behavior of both models via computing loss and accuracy on training and validation sets after each epoch. In Figure 5, we depict the loss and accuracy curves of the UNT and BCD-UNT models.

**Figure 6:**
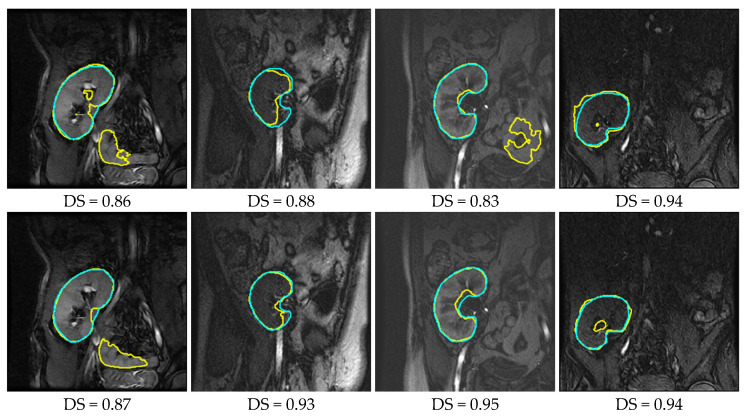
Qualitative results outlined in yellow for the UNT model (first row) and BCD-UNT model (second row) on four DCE-MRI kidney images with overlaid ground truth segmentations in cyan (DS reported below each result).

Having trained the deep UNT models, we experimentally investigated their performances on 15 DCE-MRI test datasets. We furthermore formed a set of images of low contrast, the first five time point images of each subject’s series. These images came from the pre-contrast region of the acquired sequences (refer to Figure 1); thus, it was even more challenging to delineate the kidney against the surrounding tissues. For the quantitative assessment of the segmentation accuracy, we employed the most commonly used evaluation metrics: DS, IU, and HD95% [20,21]. These metrics measured how similar the segmentation results and segmentations of the MRI expert were. Table 2 presents the segmentation accuracy (mean ± standard deviation) of the two deep UNT-based models, while sample results are displayed in Figure 6.

It is manifest in Table 2 that the two deep models have better accuracies in terms of the DS metric than those reported in Table 1 by other researchers. Moreover, the BCD-UNT model has a better segmentation performance than the baseline UNT model does. According to mean HD95% metric, the BCD-UNT model outperformed the UNT model by a margin of approximately 5.6% on all test images, rising to about 12% on the low-contrast set of images. Yet, as shown in Figure 6, both of them still suffered from some false-negative and false-positive segmentations. One way to interpret these results is that the deep models do not capture well the shape of kidneys. This can be possibly rectified by training the models on even larger datasets, which is rather difficult to realize in practice (a common problem of deep learning in medical applications). Another alternative, yet more feasible strategy is to subject the results obtained from the deep models to a subsequent refinement process. In the next section, Section 4, we propose the use of a level set-method that efficiently takes into account the kidney’s shape prior information to carry out the above, thus eliminating incorrect predictions and boosting the segmentation accuracy.

### 3.3. Ablation Experiments

The BCD-UNT model outperformed the baseline UNT for DCE-MRI kidney segmentation in our experiments. Thus, we performed another study to further investigate the BCD-UNT model’s performance with various hyper-parameters. In this study, we focused on the more important parameters for this purpose. For all experiments, we used the DS and HD95% metrics for assessing the segmentation performance on all test images as well as on the low-contrast images of the test set. First, we tested the model with two of the most common used loss functions, namely binary cross entropy (BCE), and the summation of DS and BCE (DS-BCE). Afterwards, we explored the impact of changing the dropout (DP) regularization values on the segmentation performance. Additionally, we analyzed the model’s behavior with different initial learning rates (ILRs). Quantitative results are reported in Table 3, where bold values denote the best result. Clearly, the combination of BCE, ILR = 0.0001 and DP = 0.5 achieved the best overall performance.

## 4. UNT Level Set-Based Kidney Segmentation Approach

An object’s shape is a geometrical description of the object boundary, which plays an important role in medical imaging applications, especially in segmentation tasks [37]. One key observation from our results in the previous section is that our deep neural network models did learn well to separate the kidney from the surrounding tissues based on the low-level image information, i.e., intensity information. They, however, did not learn equally well higher-level information about the shape of desired object, a task that often necessitates larger training data sets.

In this section, we extend our segmentation approach to a two-phase one, named the UNT Level Set (UNLS) approach, which integrates the developed deep BCD-UNT model with a LST method that is particularly designed to learn higher-level shape prior (SHP) information about a kidney’s shape. The employment of LST methods for this sake is motivated by their popularity in medical image segmentation problems [23,24,25,26,27,28,29,30,31,32,33,34] and their ability to embed a priori knowledge about the shape of interest in a segmentation task [22].

**Figure 7:**
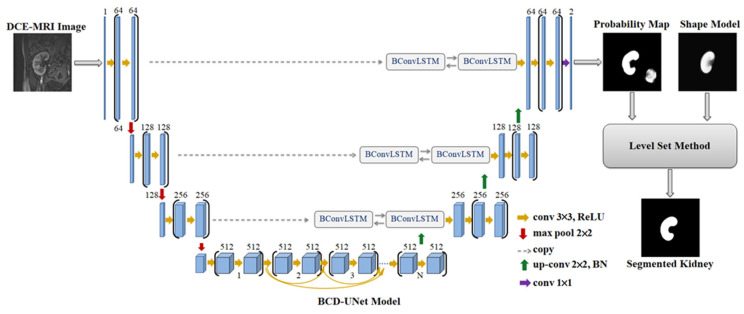
The proposed UNLS approach.

As illustrated in Figure 7, the deep BCD-UNT model generates kidney probability map for a DCE-MRI test image. Then, the obtained prior probability map is incorporated with the SHP-information into the LST method to guide the level set’s contour evolution towards the target kidney in the image. False positives and/or negatives possibly generated from the BCD-UNT model are removed at the LST phase, leading to more precise segmentation.

**Figure 8:**
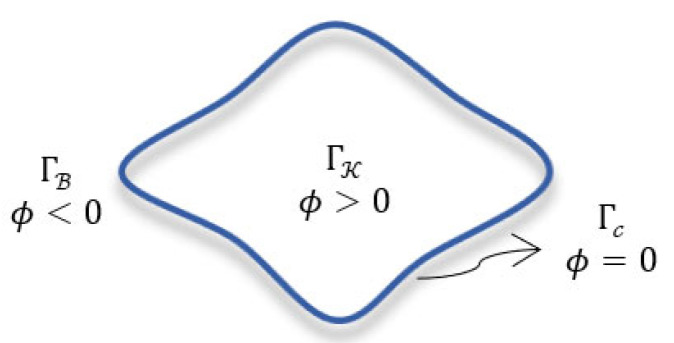
A graphical representation of the LST contour in the image domain.

Let $I_{t}$ be a DCE-MRI image to be segmented. The LST contour $\Gamma_{c}$ assigns the pixels in the image domain Γ in two disjoint regions, i.e., kidney region $\Gamma_{K}$ and background region $\Gamma_{B}$. As illustrated in Figure 8, this contour is represented by a LST function, ϕ, whose values are positive/negative for pixels in kidney/background regions and zero for the pixels on the contour itself.

The LST method accurately separates the kidney from the background by minimizing an energy functional formulated as follows:

(1) $$E\left(\phi \right)=\lambda_{1}\;L\left(\phi \right)+\lambda_{2}\;E_{u}\left(\phi \right)$$

where $\lambda_{i}$ is a positive constant coefficient and ϕ is the LST function satisfying

(2) $$\begin{array}{cc} \phi \left(x,y\right)>0, & \left(x,y\right)\in \;\Gamma_{K}\\ \phi \left(x,y\right)<0, & \left(x,y\right)\in \;\Gamma_{B}\\ \phi \left(x,y\right)=0, & \left(x,y\right)\in \;\Gamma_{c} \end{array}$$

The length term L(ϕ) in (1) ensures the smoothness of the LST contour, and is given by the following:

(3) $$L\left(\phi \right)=\overset{\;}{\underset{\Gamma }{\int }}\delta \phi_{\varepsilon }\;\left|\nabla \phi \left(x,y\right)\right|\;dx\;dy\;\;$$

where $\delta \phi_{\varepsilon }=\delta_{\varepsilon }\left(\phi \left(x,y\right)\right)$ is the Dirac delta function, the derivative of the smoothed Heaviside function, $V\phi_{\varepsilon }=V_{\varepsilon }\left(\phi \left(x,y\right)\right)$:

(4) $$V\phi_{\varepsilon }=\{\begin{array}{l} 1\;\;\;\;\;\;\;\;\;\;\;\;\;\;\;\;\;\;\;\;\;\;\;\;\;\;\;\;\;\;\;\;\;\;\;\;\;\;\;\;\;\;\;\;\;\;\;\;\;\;\;\;\;\;\;\;\;\;\;\;\;\;\;\;\;\;\;\;\;\;\phi \left(x,y\right)>\varepsilon \;\;\;\;\;\\ \frac{1}{2}+\frac{\phi \left(x,y\right)}{2\varepsilon }+\frac{1}{2\pi }\sin\left(\frac{\pi \phi \left(x,y\right)}{\varepsilon }\right)\;\;\;\;\;\;\;\;\;\;\;\;\;\;\;-\varepsilon \leq \phi \left(x,y\right)\leq \varepsilon \;\;\;\;\;\\ 0\;\;\;\;\;\;\;\;\;\;\;\;\;\;\;\;\;\;\;\;\;\;\;\;\;\;\;\;\;\;\;\;\;\;\;\;\;\;\;\;\;\;\;\;\;\;\;\;\;\;\;\;\;\;\;\;\;\;\;\;\;\;\;\;\;\;\;\;\;\;\phi \left(x,y\right)<-\varepsilon \;\;\;\;\; \end{array}$$

(5) $$\delta \phi_{\varepsilon }=\{\begin{array}{l} 0\;\;\;\;\;\;\;\;\;\;\;\;\;\;\;\;\;\;\;\;\;\;\;\;\;\;\;\;\;\;\;\;\;\;\;\;\;\;\;\;\;\;\;\;\;\;\;\;\;\;\;\;\;\;\;\;\;\;\;\;\;\;\;\;\;\;\;\;\left|\phi \left(x,y\right)\right|>\varepsilon \;\;\;\;\;\\ \frac{1}{2\varepsilon }+\frac{1}{2\varepsilon }\cos\left(\frac{\pi \phi \left(x,y\right)}{\varepsilon }\right)\;\;\;\;\;\;\;\;\;\;\;\;\;\;\;\;\;\;\;\;\;\;\;\;\;\;\;\;\;\;\;\;\;\;\left|\phi \left(x,y\right)\right|\leq \varepsilon \;\;\;\;\; \end{array}$$

where ε represents the regularization coefficient.

The energy functional $E_{u}\left(\phi \right)$ in (1) depends mainly on the input image, plays a leading role in directing the LST contour to the desired kidney boundary in the evolution procedure, and is denoted as follows:

(6) $$E_{u}\left(\phi \right)=\overset{\;}{\underset{\Gamma }{\int }}V\phi_{\varepsilon }\;U_{B}\left(x,y\right)\;\;P_{B}\left(x,y\right)\;dx\;dy\;+\overset{\;}{\underset{\Gamma }{\int }}\left(1-V\phi_{\varepsilon }\right)\;U_{K}\left(x,y\right)\;\;P_{K}\left(x,y\right)\;dx\;dy\;$$

where $U_{L}\in \left[0,1\right]$ represents the kidney/background probability map obtained from the already-trained BCD-UNT model satisfying $\overset{\;}{\underset{L}{\sum }}U_{L}\left(x,y\right)=1$, i.e.,., $U_{L}\left(x,y\right)\;$denotes the probability of the pixel (x,y) belonging to the kidney (i.e., L=K) or background (i.e., L=B). $P_{L}$ is the kidney/background probabilistic SHP model built embracing the Bayesian parameter estimation method, whose details are described next. Differentiating (1) with respect to ϕ leads to the corresponding gradient descent formula:

(7) $$\frac{\partial \phi }{\partial t}=-\frac{\partial E}{\partial \phi }=\delta \phi_{\varepsilon }\left[\;\lambda_{1}\mathrm{div}\left(\frac{\nabla \phi \left(x,y\right)}{\left|\nabla \phi \left(x,y\right)\right|}\right)+\lambda_{2}\;U_{K}\;P_{K}-\lambda_{2}\;U_{B}\;P_{B}\;\right]$$

Additionally, eventually, the LST contour is iteratively evolved according to

(8) $$\phi_{n+1}=\phi_{n}+\tau \;\frac{\partial \phi_{n}}{\partial t}$$

where τ>0 and n is the time step. Notably, employing the regularized form of the Heaviside and Dirac delta functions increases the efficiency of numerical calculation and assures the convergence toward the global minimum for the functional in (1) starting from a randomly initialized contour [22].

### 4.1. Probabilistic Shape Model

Human kidneys often have well-known shapes. Consequently, incorporating discriminative information such as kidney shape can drastically aid in achieving more robust segmentation performance. Several approaches have been used to construct statistical shape models in the literature. Among them, the first-order shape method [28,29,30,31] is considered one of the most often-used methods. The main drawback of this method is that, when a pixel is observed as a kidney in all images, it assumes that the pixel’s probability of being a kidney is 100%, that the background probability is 0% and vice versa, which may distort the segmentation results.

**Figure 9:**
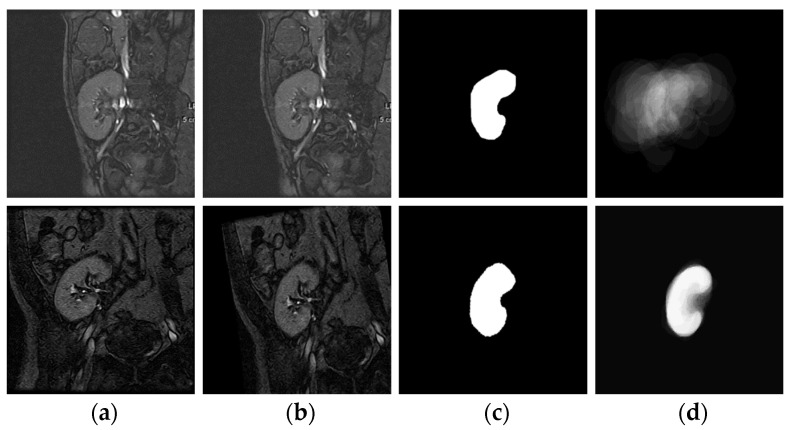
Construction of the SHP model adopting the Bayesian parameter estimation method. Columns (**a**,**b**) show non-registered and registered DCE-MRI kidney images. Column (**c**) shows ground-truth segmentations. Column (**d**) depicts the SHP-model built before (top) and after (bottom) affine registration.

To tackle this drawback, we here embrace a statistically efficient Bayesian parameter estimation method [38] for SHP model formulation. As illustrated in Figure 9, the SHP model is basically built from a diverse set of kidney images as follows. First, one of these images is chosen as a reference. Then, all other images are affinely registered [39] to the reference image. Finally, experienced clinicians manually segment the kidneys in the co-aligned images.

If the pixel (x,y) appears as kidney in a number of images, while in others it appears as background, the empirical pixel-wise probability of both labels (kidney and background) is computed from the following [37,38]:

(9) $$P_{L}\left(x,y\right)=\left[\frac{N_{L}\left(x,y\right)+\beta }{\;\;N+\beta \;O\left(x,y\right)\;}\right]\;\left[\frac{N}{\;N+l-O\left(x,y\right)\;}\right]$$

where l=2 refers to the count of all prospective labels and N denotes the number of co-aligned labeled images. O(x,y) represents how many labels have been observed, in which case, O(x,y)=2. $N_{L}\left(x,y\right)$ stands for how frequently the label L has appeared and β is a positive pseudo-count. It should be noted that $P_{L}\left(x,y\right)\in \left[0,1\right],$ where $\sum_{L}P_{L}\left(x,y\right)=1$. Alternatively, in case the pixel (x,y) is either classed as kidney or background in all training series, the probability of the label appearing in the scene is calculated using the above formula, while the probability of the label that has not been seen is computed from the following:

(10) $$P_{L}\left(x,y\right)=\left[\frac{1}{\;\;l-O\left(x,y\right)\;}\right]\;\left[1-\frac{N}{\;N+l-O\left(x,y\right)\;}\right]$$

where O(x,y) will be 1 since the kidney label only is observed. According to this, as depicted in Figure 9, a more distinctive shape model is built.

### 4.2. Results

We, in this section, conduct thorough experiments to assess the proposed UNLS approach’s performance. The statistical SHP model is explicitly learned from a varying set of ground truth kidneys from 30 different patients. The optimal values for the proposed approach’s parameters are experimentally chosen and fixed as $\lambda_{1}=6$, $\lambda_{2}=6$, ε=1.5, τ=0.8, and β=1 over all experiments without any further tuning. Table 4 reports the quantitative evaluation results of the proposed approach over all the test images and the set of low-contrast images.

**Figure 10:**
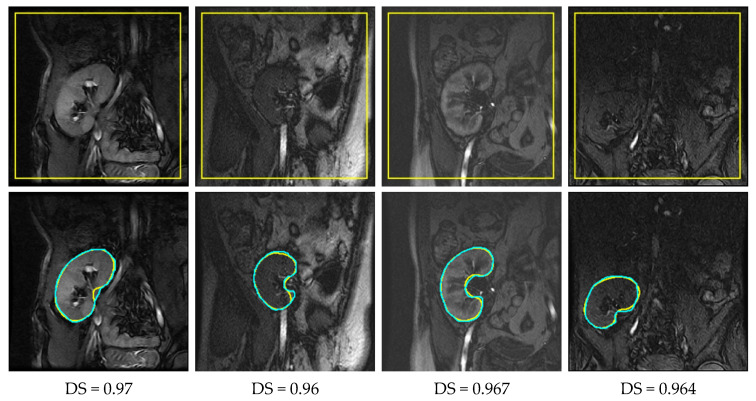
Segmentation results of UNLS for the same DCE-MRI images shown in Figure 6. First row depicts the initial LST contour. Second row depicts segmented kidneys in yellow and the ground truth kidneys in cyan. Computed DS values are shown below each image.

Results in both Table 2 and Table 4 substantiate the considerable increase in the performance of the proposed approach over that of the two deep models. UNLS has higher mean DS values than the UNT and BCD-UNT models do, while it has considerably lower mean HD95% values. More specifically, it achieves a higher segmentation performance than the UNT model does, in terms of mean HD95%, with an improvement of 8.71 mm and up to 17.3 mm on low-contrast images. As for the BCD-UNT model, UNLS remarkably outperforms it by about 3.01 mm, reaching 14.4 mm on the low-contrast set. This in turn confirms that incorporating a kidney SHP-information significantly reduces the false positive/negative rates and boosts segmentation performance. Furthermore, the lower standard deviations of all the reported evaluation metrics firmly demonstrate the highly consistent performance of the proposed approach compared to that of the two models. Figure 10 visually portrays the segmentation results of the proposed approach.

It is evident from Figure 6 that the UNT and BCD-UNT models incorrectly identified background pixels as kidneys, while in other cases they were confused by tissues inside the kidney region. As a consequence, they generated inaccurate segmentation results. The proposed UNLS approach, in contrast, achieved high segmentation accuracy as revealed from Figure 10. We deliberately initialized the LST contour far from the kidney position in all conducted experiments (as depicted in the first row of Figure 10). Nevertheless, UNLS accurately segmented the kidneys out from the background and gave more accurate and reliable results.

**Figure 11:**
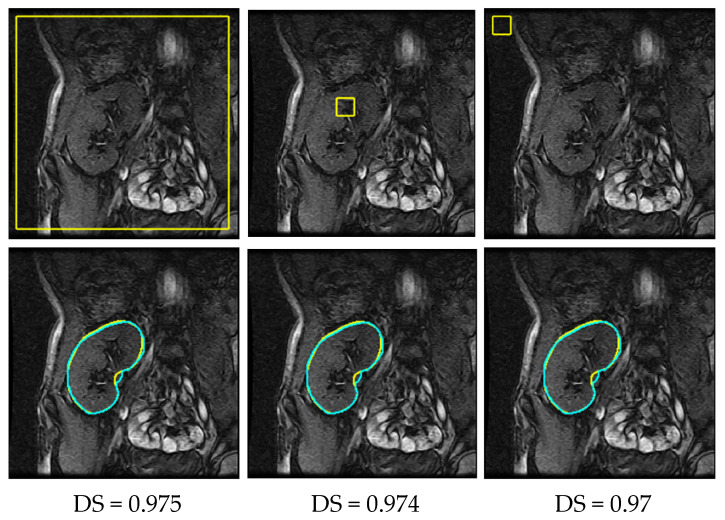
The proposed approach’s performance with different LST contour initializations. The first row shows the contour initializations on a sample DCE-MRI image, while the second demonstrates the segmentation results in yellow with the ground truths superimposed over the images in cyan. Computed DS values are attached below each result.

We then conducted several experiments to study the impact of LST contour initialization on the efficacy of UNLS. Figure 11 reveals the segmentation results obtained using UNLS with different initial contours. Apparently, the LST contour consistently converged to the kidney boundary in all cases. This confirms that the proposed approach’s performance was unaffected by changing where the contour was initialized in the image.

We further demonstrate the competence of UNLS by comparing it with numerous recent LST-based methods: shape-based (SLST) [23], vector level sets (VLST) [24], FCMLS [30], PBPSFL [31], PSFL [32], FML [33], and JSRL [34]. A quantitative comparison is presented in Table 5 of the same two test sets of all images and low-contrast images. The results clearly demonstrate that UNLS explicitly achieves promising performance compared to its counterparts. The approach outperforms almost all of these methods. The exceptions are the PSFL and FML methods, which surpass in performance the proposed UNLS approach. Both are based on a LST method that employs more sophisticated statistical mechanisms: global and patient-specific shape statistics in the PSFL method [32] and Markov random field modeling in the FML method [33]. Our underway research is directed towards investigating the employment of similar mechanisms in our proposed approach to improve its performance even further.

## 5. Conclusions

Kidney segmentation from DCE-MRI is an important step in the functional phase of renal function assessment. We have proposed in this paper novel and automated approaches for accurate kidney segmentation from DCE-MRI. First, we investigated a deep learning approach for this task based on the popular UNT architecture and one of its successful variants, BCD-UNT. The two deep models were trained on a training dataset of more than 40 thousand images, and demonstrated segmentation performances on the test dataset that were better than those of several already-reported deep approaches in the literature.

Our analysis of the obtained results revealed that the deep models learn well a kidney’s low-level intensity information while they do not capture well higher-level kidney shape information. Learning such high-level information would require larger data sets, which is a typical problem in the medical applications of deep learning. To rectify this situation, we proposed a two-phase approach. The first phase of UNLS depends on the BCD-UNT deep model to produce a kidney probability map. In the second phase, a LST method is formulated to minimize a new energy functional that considers both the predicted probability map and the kidney’s shape prior information, thus generating the final kidney segmentation.

The paper’s contributions are summarized as follows:

- It integrates the merits of deep neural networks and the LST method, for the first time, to accomplish this task.
- It proposes a new energy functional incorporating a kidney/background probability map generated from a deep neural model and shape prior information to steer the LST contour towards the target kidney.
- It employs an efficient Bayesian parameter estimation method in the computation of SHP information, which can statistically handle the cases of unobserved kidney/background pixels in constructing the shape model.

We have evaluated the proposed UNLS approach on DCE-MRI datasets from 45 patients. It achieved a significant increase in performance, providing a HD95% score of 1.54 mm with a performance boost of about 8.76 mm and 3 mm in comparison with that of the UNT and BCD-UNT deep models, respectively. We have also compared the approach against various recent LST-based methods. Our experiments affirmed the potential and robust performance using the proposed approach in segmenting kidneys from DCE-MRI data.

Despite the experimentally demonstrated good performance of the proposed approach, it still has some limitations. First, deep learning models typically require a large amount of data for network training, which is often difficult to obtain in the medical field. Second, employing the shape prior information of kidney imposes the requirement of a registration operation to align the image that needs to be segmented to a pre-constructed shape model. In our new approach, this operation is performed before the kidney segmentation task. The main drawback of this is that errors occur in the registration step that significantly affect the segmentation performance. Third, as do all the level set-based methods, our new approach depends on the principle of partial differential equations that contain weighting parameters. All these weighting parameters require proper setting. In our experiments, the values of these parameters were experimentally chosen and fixed throughout all conducted experiments without further tuning. Our ongoing research efforts are directed towards finding solutions to alleviate these limitations. Moreover, seeking a further performance boost, we plan to investigate adopting more advanced shape statistics, such as the global and patient-specific shape statistics of [32], in the proposed UNLS.

Another direction worthy of further investigation is the adoption of the proposed approach for other related diseases, such as polycystic kidney disease. Segmentation of kidneys infected with this disease from MRI scans poses several challenges due to the large distortions and structural abnormalities, which we plan to address in another follow-up work.

## Figures and Tables

**Table 1:** Summary of related deep learning-based methods for MRI kidney segmentation.

Reference	Method	Number of Patients/Modality		DS
Lundervold et al. [6]	ConvNets	20	DCE-MRIs	0.87/85 Left/Right
Haghighi et al. [7]	Two cascaded 3D UNTs	30	Pediatric DCE-MRIs	0.91 ± 0.03 Normal 0.83 ± 0.03 Abnormal
Bevilacqua et al. [9]	ConvNets (VGG-16)	18	T2-Weighted MRIs	0.85
Brunetti et al. [10]	ConvNets With Genetic Algorithm	18	T2-Weighted MRIs	0.91
Milecki et al. [11]	ConvNets With Thresholding	32	DCE and T2 MRIs	0.89 ± 0.0317
Isensee et al. [12]	nnUNT	40	T1-DUAL IP/OP and T2-SPIR MRIs	0.94 ± 0.0159
Asaturyan et al. [14]	3D Rb-UNT	60	4D DCE-MRIs	0.88 ± 0.064
Goyal et al. [16]	Mask R-CNN	100	MRIs	0.90 ± 0.041

**Table 2:** Segmentation performance of the deep UNT and BCD-UNT models.

Method	All DCE-MRIs			Low-Contrast DCE-MRIs		
	DS	IU	HD95%	DS	IU	HD95%
UNT	0.940 ± 0.04	0.89 ± 0.07	10.3 ± 23.8	0.88 ± 0.07	0.77 ± 0.13	19.9 ± 28.8
BCD-UNT	**0.942 ± 0.04**	**0.89 ± 0.06**	**4.6 ± 12.4**	**0.90 ± 0.06**	**0.82 ± 0.09**	**7.9 ± 12.3**

**Table 3:** Segmentation performance of BCD-UNT model with different hyper-parameters values.

Exp.	Loss Function	ILR	DP	All DCE-MRIs			Low-Contrast DCE-MRIs		
				DS	IU	HD95%	DS	IU	HD95%
1	BCE	0.0001	0.1	0.929 ± 0.11	0.88 ± 0.13	5.77 ± 16.9	0.72 ± 0.28	0.62 ± 0.29	26.9 ± 32.9
2	BCE	0.0001	0.5	**0.942 ± 0.04**	**0.89 ± 0.06**	**4.62 ± 12.4**	**0.90 ± 0.057**	**0.82 ± 0.09**	**7.89 ± 12.3**
3	BCE	0.0001	0.8	0.946 ± 0.05	0.90 ± 0.08	8.57 ± 21.2	0.92 ± 0.06	0.85 ± 0.09	13.4 ± 23.6
4	DS-BCE	0.0001	0.5	0.94 ± 0.053	0.89 ± 0.08	7.24 ± 17.9	0.88 ± 0.13	0.89 ± 0.08	12.2 ± 20.6
5	BCE	0.001	0.5	0.92 ± 0.068	0.86 ± 0.098	16.27 ± 25.5	0.81 ± 0.15	0.70 ± 0.18	26.9 ± 29.4
6	BCE	0.0005	0.5	0.90 ± 0.25	0.83 ± 0.24	25.57 ± 31.7	0.87 ± 0.26	0.79 ± 0.25	49.1 ± 31.8

**Table 4:** Segmentation performance of the proposed UNLS approach.

Method	All DCE-MRIs			Low-Contrast DCE-MRIs		
	DS	IU	HD95%	DS	IU	HD95%
UNLS	0.952 ± 0.02	0.91 ± 0.03	1.54 ± 1.6	0.93 ± 0.039	0.87 ± 0.06	2.6 ± 2.8

**Table 5:** Comparison of the segmentation performance using UNLS and existing LST-based methods.

Method	All DCE-MRIs			Low-Contrast DCE-MRIs		
	DS	IU	HD95%	DS	IU	HD95%
VLST [24]	0.91 ± 0.074	0.84 ± 0.1	3.4 ± 6.71	0.93 ± 0.06	0.87 ± 0.09	2.00 ± 4.44
SLST [23]	0.92 ± 0.037	0.85 ± 0.06	2.4 ± 1.4	0.93 ± 0.033	0.87 ± 0.06	2.05 ± 1.4
FCMLS [30]	0.94 ± 0.035	0.89 ± 0.04	1.4 ± 2.0	0.90 ± 0.06	0.82 ± 0.09	4.7 ± 4.6
PBPSFL [31]	0.95 ± 0.025	0.90 ± 0.036	1.09 ± 1.8	0.93 ± 0.04	0.87 ± 0.07	2.8 ± 3.96
FML [33]	0.96 ± 0.017	0.925 ± 0.03	0.68 ± 1.19	0.935 ± 0.037	0.88 ± 0.06	2.23 ± 3.6
PSFL [32]	0.957 ± 0.016	0.93 ± 0.019	0.80 ± 1.03	0.95 ± 0.014	0.90 ± 0.026	0.85 ± 0.76
JSRL [34]	0.954 ± 0.026	0.91 ± 0.04	0.81 ± 1.3	0.92 ± 0.07	0.86 ± 0.09	2.6 ± 3.8
UNLS	0.952 ± 0.02	0.91 ± 0.03	1.54 ± 1.6	0.93 ± 0.039	0.87 ± 0.06	2.6 ± 2.8

## Data Availability

Data are available upon reasonable request to the corresponding author.
